# Ethyl 2-[2-(3-meth­oxy­phen­yl)hydrazinyl­idene]-3-oxobutano­ate

**DOI:** 10.1107/S1600536811039444

**Published:** 2011-09-30

**Authors:** Hoong-Kun Fun, Safra Izuani Jama Asik, Ibrahim Abdul Razak, Shobhitha Shetty, Balakrishna Kalluraya

**Affiliations:** a X-ray Crystallography Unit, School of Physics, Universiti Sains Malaysia, 11800 USM, Penang, Malaysia; b Department of Studies in Chemistry, Mangalore University, Mangalagangotri, Mangalore 574 199, India

## Abstract

The title compound, C_13_H_16_N_2_O_4_, is approximately planar (r.m.s. deviation = 0.065 Å for the 19 non-H atoms). An intra­molecular N—H⋯O hydrogen bond generates an *S*(6) ring motif and the mol­ecule adopts an *E* conformation with respect to the central C=N double bond. In the crystal, pairs of inter­molecular C—H⋯O hydrogen bonds link adjacent mol­ecules into inversion dimers. The crystal structure also features weak C—H⋯π inter­actions.

## Related literature

For the biological activity of oxobutano­ate derivatives, see: Billington *et al.* (1979 ▶); Stancho *et al.* (2008 ▶); For the biological activity of pyrazole derivatives, see: Rai *et al.* (2008 ▶); Girisha *et al.* (2010 ▶); Isloor *et al.* (2009 ▶). For hydrogen-bond motifs, see: Bernstein *et al.* (1995 ▶). For a related structure, see: Fun *et al.* (2011 ▶).
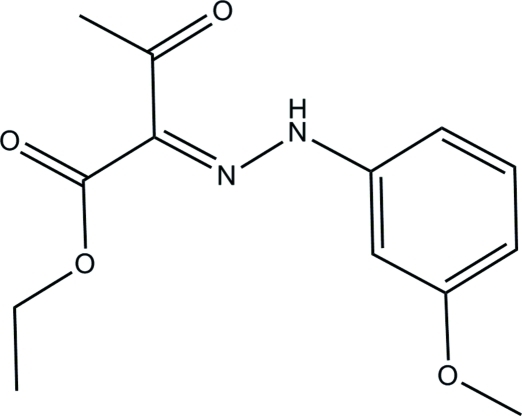


## Experimental

### 

#### Crystal data

C_13_H_16_N_2_O_4_
*M* *_r_* = 264.28
Triclinic, 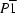

*a* = 5.7796 (4) Å
*b* = 7.4691 (5) Å
*c* = 16.9842 (11) Å
α = 77.956 (2)°
β = 89.394 (2)°
γ = 72.547 (2)°
*V* = 682.97 (8) Å^3^
*Z* = 2
Mo *K*α radiation
μ = 0.10 mm^−1^
*T* = 296 K
0.53 × 0.36 × 0.25 mm

#### Data collection

Bruker APEX DUO CCD diffractometer
Absorption correction: multi-scan (*SADABS*; Bruker, 2009 ▶) *T*_min_ = 0.942, *T*_max_ = 0.977
11969 measured reflections
3108 independent reflections
2419 reflections with *I* > 2σ(*I*)
*R*_int_ = 0.021

#### Refinement

*R*[*F* ^2^ > 2σ(*F* ^2^)] = 0.050
*wR*(*F* ^2^) = 0.180
*S* = 1.04
3108 reflections
179 parameters
H atoms treated by a mixture of independent and constrained refinement
Δρ_max_ = 0.22 e Å^−3^
Δρ_min_ = −0.20 e Å^−3^

### 

Data collection: *APEX2* (Bruker, 2009 ▶); cell refinement: *SAINT* (Bruker, 2009 ▶); data reduction: *SAINT*; program(s) used to solve structure: *SHELXTL* (Sheldrick, 2008 ▶); program(s) used to refine structure: *SHELXTL*; molecular graphics: *SHELXTL*; software used to prepare material for publication: *SHELXTL* and *PLATON* (Spek, 2009 ▶).

## Supplementary Material

Crystal structure: contains datablock(s) global, I. DOI: 10.1107/S1600536811039444/hb6413sup1.cif

Structure factors: contains datablock(s) I. DOI: 10.1107/S1600536811039444/hb6413Isup2.hkl

Supplementary material file. DOI: 10.1107/S1600536811039444/hb6413Isup3.cml

Additional supplementary materials:  crystallographic information; 3D view; checkCIF report

## Figures and Tables

**Table 1:** Hydrogen-bond geometry (Å, °) *Cg*1 is the centroid of the C1–C6 ring.

*D*—H⋯*A*	*D*—H	H⋯*A*	*D*⋯*A*	*D*—H⋯*A*
N1—H1*N*1⋯O3	0.87 (2)	1.87 (2)	2.5629 (18)	135.3 (18)
C5—H5*A*⋯O3^i^	0.93	2.54	3.4389 (19)	164
C13—H13*C*⋯O4^ii^	0.96	2.58	3.219 (3)	124
C12—H12*B*⋯*Cg*1^iii^	0.96	2.82	3.6422 (17)	145

Symmetry codes: (i) 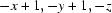; (ii) 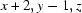; (iii) 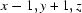.

**Fig. 1.**
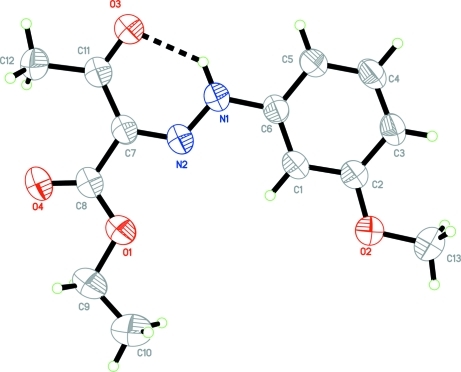
The structure of the title compound, showing 50% probability displacement ellipsoids. Hydrogen atoms are shown as spheres of arbitrary radius.

**Fig. 2.**
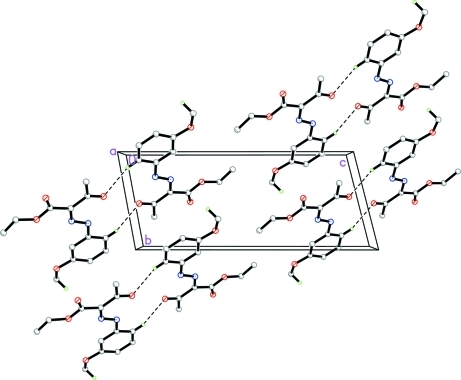
The crystal packing, viewed along the a-axis, showing the formation of dimers. Hydrogen atoms that not involved in hydrogen bonding (dashed lines) are omitted for clarity.

## supplementary crystallographic information

### Comment

Derivatives of oxobutanoates are biologically important. 4-Methylthio-2-oxobutanoate was identified in the culture fluids of a range of bacteria, *e.g*. the yeast *Saccharomyces cerevisiae* and the fungus *Penicillium digitatum* (Billington *et al.*, 1979). Some oxobutanoates exhibit cytotoxic properties (Stancho *et al.*, 2008). Pyrazole derivatives are well established in the literatures as important biologically effective heterocyclic compounds (Rai *et al.*, 2008). These derivatives are the subject of many research studies due to their widespread pharmacological activities such as anti-inflammatory (Girisha *et al.*, 2010), antipyretic, antimicrobial (Isloor *et al.*, 2009), and antiviral activities. The widely prescribed anti-inflammatory pyrazole derivatives, celecoxib and deracoxib, are selective COX-2 inhibitors with reduced ulcerogenic side effects. The title compound (I), ethyl-2-[2-(3-methoxyphenyl)hydrazinylidene]-3-oxobutanoate, is a key intermediate in the preparation of pyrazole derivative. Condensation of oxobutanoate with thiosemicarbazide in glacial acetic acid medium gave the required pyrazole derivatives.

In the title compound of (I) (Fig. 1), an intramolecular N1—H1N1···O3 hydrogen bond (Table 1) generates a six–membered ring producing an S(6) ring motif (Bernstein *et al.*, 1995). The molecule adopts an *E*-configuration with respect to the central C7═N2 double bond. Similar configuration was also reported in the crystal structure of (Fun *et al.*, 2011).

In the crystal structure of (Fig. 2), intermolecular C5—H5A···O3 and C13—H13C···O4 hydrogen bonds link the molecules into dimers. The crystal structure is further stabilizied by weak C—H···π interactions (Table 1) with distance of 3.6422 (17) Å involving the C1–C6 (centroid *Cg*1) ring.

### Experimental

The title compound was prepared by dissolving 3-methoxy aniline (0.01 mol) in dilute hydrochloric acid (10 ml) and cooled to 0 °C in an ice bath. To this, a cold solution of sodium nitrite (0.02 mol) was added. The resulting diazonium salt solution was filtered into a cold solution of ethyl acetoacetate (0.05 mol) and sodium acetate in ethanol. The separated yellow solid was filtered, washed with water and recrystallized from ethanol to yield yellow blocks of (I).

### Refinement

Atom H1N1 was located in a difference Fourier map and refined freely with N–H = 0.871 (19) Å. The remaining H atoms were positioned geometrically with C–H = 0.93–0.97 Å. The *U*_iso_ values were constrained to be 1.5*U*_eq_ of the carrier atom for methyl H atoms and 1.2*U*_eq_ for the remaining H atoms. A rotating group model was used for the methyl groups.

### Crystal data

**Table:**

C_13_H_16_N_2_O_4_	*Z* = 2
*M**_r_* = 264.28	*F*(000) = 280
Triclinic, *P*1	*D*_x_ = 1.285 Mg m^−^^3^
Hall symbol: -P 1	Mo *K*α radiation, λ = 0.71073 Å
*a* = 5.7796 (4) Å	Cell parameters from 5306 reflections
*b* = 7.4691 (5) Å	θ = 2.9–31.0°
*c* = 16.9842 (11) Å	µ = 0.10 mm^−^^1^
α = 77.956 (2)°	*T* = 296 K
β = 89.394 (2)°	Block, yellow
γ = 72.547 (2)°	0.53 × 0.36 × 0.25 mm
*V* = 682.97 (8) Å^3^	

### Data collection

**Table:**

Bruker APEX DUO CCD diffractometer	3108 independent reflections
Radiation source: fine-focus sealed tube	2419 reflections with *I* > 2σ(*I*)
graphite	*R*_int_ = 0.021
φ and ω scans	θ_max_ = 27.5°, θ_min_ = 1.2°
Absorption correction: multi-scan (*SADABS*; Bruker, 2009)	*h* = −7→7
*T*_min_ = 0.942, *T*_max_ = 0.977	*k* = −9→9
11969 measured reflections	*l* = −22→22

### Refinement

**Table:**

Refinement on *F*^2^	Primary atom site location: structure-invariant direct methods
Least-squares matrix: full	Secondary atom site location: difference Fourier map
*R*[*F*^2^ > 2σ(*F*^2^)] = 0.050	Hydrogen site location: inferred from neighbouring sites
*wR*(*F*^2^) = 0.180	H atoms treated by a mixture of independent and constrained refinement
*S* = 1.04	*w* = 1/[σ^2^(*F*_o_^2^) + (0.1108*P*)^2^ + 0.0852*P*] where *P* = (*F*_o_^2^ + 2*F*_c_^2^)/3
3108 reflections	(Δ/σ)_max_ < 0.001
179 parameters	Δρ_max_ = 0.22 e Å^−^^3^
0 restraints	Δρ_min_ = −0.20 e Å^−^^3^

### Special details

**Table:**

Geometry. All esds (except the esd in the dihedral angle between two l.s. planes) are estimated using the full covariance matrix. The cell esds are taken into account individually in the estimation of esds in distances, angles and torsion angles; correlations between esds in cell parameters are only used when they are defined by crystal symmetry. An approximate (isotropic) treatment of cell esds is used for estimating esds involving l.s. planes.
Refinement. Refinement of F^2^ against ALL reflections. The weighted R-factor wR and goodness of fit S are based on F^2^, conventional R-factors R are based on F, with F set to zero for negative F^2^. The threshold expression of F^2^ > 2sigma(F^2^) is used only for calculating R-factors(gt) etc. and is not relevant to the choice of reflections for refinement. R-factors based on F^2^ are statistically about twice as large as those based on F, and R- factors based on ALL data will be even larger.

### Fractional atomic coordinates and isotropic or equivalent isotropic displacement parameters (Å^2^)

**Table:**

	*x*	*y*	*z*	*U*_iso_*/*U*_eq_	
O1	0.1556 (2)	0.3160 (2)	0.33318 (7)	0.0707 (4)	
O2	1.1429 (2)	−0.1952 (2)	0.34750 (7)	0.0743 (4)	
O3	0.2371 (2)	0.53420 (17)	0.05938 (7)	0.0633 (3)	
O4	−0.1755 (2)	0.5181 (2)	0.26261 (8)	0.0777 (4)	
N1	0.5632 (2)	0.27108 (17)	0.15513 (8)	0.0466 (3)	
N2	0.4082 (2)	0.29646 (16)	0.21110 (7)	0.0465 (3)	
C1	0.8606 (3)	0.0293 (2)	0.25233 (9)	0.0506 (4)	
H1A	0.7505	0.0426	0.2927	0.061*	
C2	1.0931 (3)	−0.0981 (2)	0.26917 (9)	0.0522 (4)	
C3	1.2578 (3)	−0.1225 (2)	0.20927 (10)	0.0542 (4)	
H3A	1.4129	−0.2093	0.2208	0.065*	
C4	1.1857 (3)	−0.0148 (2)	0.13213 (9)	0.0553 (4)	
H4A	1.2943	−0.0305	0.0915	0.066*	
C5	0.9560 (3)	0.1162 (2)	0.11373 (9)	0.0505 (3)	
H5A	0.9107	0.1890	0.0617	0.061*	
C6	0.7950 (2)	0.13607 (19)	0.17489 (8)	0.0441 (3)	
C7	0.1888 (2)	0.41968 (19)	0.19492 (8)	0.0461 (3)	
C8	0.0353 (3)	0.4262 (2)	0.26510 (9)	0.0528 (4)	
C9	0.0175 (4)	0.3139 (4)	0.40468 (11)	0.0865 (7)	
H9A	−0.1166	0.2641	0.3986	0.104*	
H9B	−0.0475	0.4429	0.4141	0.104*	
C10	0.1857 (5)	0.1891 (5)	0.47226 (13)	0.1126 (10)	
H10A	0.0990	0.1788	0.5207	0.169*	
H10B	0.3132	0.2433	0.4792	0.169*	
H10C	0.2545	0.0638	0.4609	0.169*	
C11	0.0984 (3)	0.5402 (2)	0.11476 (9)	0.0487 (3)	
C12	−0.1580 (3)	0.6703 (2)	0.09875 (10)	0.0597 (4)	
H12A	−0.1847	0.7315	0.0426	0.090*	
H12B	−0.1853	0.7663	0.1305	0.090*	
H12C	−0.2683	0.5963	0.1129	0.090*	
C13	1.3843 (3)	−0.3131 (4)	0.37051 (12)	0.0847 (6)	
H13A	1.3949	−0.3739	0.4266	0.127*	
H13B	1.4920	−0.2357	0.3616	0.127*	
H13C	1.4297	−0.4096	0.3389	0.127*	
H1N1	0.517 (3)	0.341 (3)	0.1067 (12)	0.065 (5)*	

### Atomic displacement parameters (Å^2^)

**Table:**

	*U*^11^	*U*^22^	*U*^33^	*U*^12^	*U*^13^	*U*^23^
O1	0.0532 (7)	0.0947 (9)	0.0492 (6)	−0.0042 (6)	0.0115 (5)	−0.0101 (6)
O2	0.0537 (7)	0.0919 (9)	0.0512 (6)	0.0048 (6)	0.0072 (5)	0.0013 (6)
O3	0.0516 (6)	0.0726 (7)	0.0522 (6)	−0.0059 (5)	0.0090 (5)	−0.0036 (5)
O4	0.0511 (7)	0.0915 (9)	0.0686 (8)	0.0058 (6)	0.0189 (5)	−0.0105 (6)
N1	0.0392 (6)	0.0488 (6)	0.0478 (6)	−0.0087 (5)	0.0078 (5)	−0.0093 (5)
N2	0.0400 (6)	0.0487 (6)	0.0508 (6)	−0.0117 (5)	0.0082 (5)	−0.0138 (5)
C1	0.0434 (7)	0.0565 (8)	0.0474 (7)	−0.0094 (6)	0.0133 (6)	−0.0106 (6)
C2	0.0459 (8)	0.0560 (8)	0.0482 (8)	−0.0087 (6)	0.0081 (6)	−0.0073 (6)
C3	0.0406 (7)	0.0566 (8)	0.0602 (9)	−0.0061 (6)	0.0114 (6)	−0.0142 (6)
C4	0.0468 (8)	0.0621 (9)	0.0554 (8)	−0.0128 (6)	0.0190 (6)	−0.0158 (6)
C5	0.0471 (8)	0.0526 (8)	0.0486 (7)	−0.0124 (6)	0.0115 (6)	−0.0086 (6)
C6	0.0394 (7)	0.0429 (6)	0.0492 (7)	−0.0108 (5)	0.0080 (5)	−0.0112 (5)
C7	0.0409 (7)	0.0474 (7)	0.0493 (7)	−0.0105 (5)	0.0078 (5)	−0.0134 (6)
C8	0.0458 (7)	0.0558 (8)	0.0533 (8)	−0.0087 (6)	0.0088 (6)	−0.0145 (6)
C9	0.0712 (12)	0.1166 (17)	0.0559 (10)	−0.0087 (11)	0.0213 (9)	−0.0142 (10)
C10	0.1045 (18)	0.146 (2)	0.0604 (13)	−0.0132 (17)	0.0132 (12)	−0.0019 (14)
C11	0.0444 (7)	0.0495 (7)	0.0518 (8)	−0.0124 (6)	0.0054 (6)	−0.0127 (6)
C12	0.0452 (8)	0.0624 (9)	0.0617 (9)	−0.0073 (6)	0.0031 (6)	−0.0054 (7)
C13	0.0576 (10)	0.1074 (16)	0.0643 (11)	0.0002 (10)	−0.0048 (8)	−0.0013 (10)

### Geometric parameters (Å, °)

**Table:**

O1—C8	1.3321 (19)	C5—C6	1.3885 (19)
O1—C9	1.446 (2)	C5—H5A	0.9300
O2—C2	1.3635 (19)	C7—C11	1.469 (2)
O2—C13	1.416 (2)	C7—C8	1.479 (2)
O3—C11	1.2286 (18)	C9—C10	1.471 (3)
O4—C8	1.2018 (18)	C9—H9A	0.9700
N1—N2	1.3029 (16)	C9—H9B	0.9700
N1—C6	1.4077 (17)	C10—H10A	0.9600
N1—H1N1	0.871 (19)	C10—H10B	0.9600
N2—C7	1.3159 (17)	C10—H10C	0.9600
C1—C6	1.379 (2)	C11—C12	1.500 (2)
C1—C2	1.386 (2)	C12—H12A	0.9600
C1—H1A	0.9300	C12—H12B	0.9600
C2—C3	1.388 (2)	C12—H12C	0.9600
C3—C4	1.381 (2)	C13—H13A	0.9600
C3—H3A	0.9300	C13—H13B	0.9600
C4—C5	1.385 (2)	C13—H13C	0.9600
C4—H4A	0.9300		
			
C8—O1—C9	115.96 (13)	O1—C8—C7	112.55 (12)
C2—O2—C13	117.72 (13)	O1—C9—C10	106.80 (17)
N2—N1—C6	119.45 (12)	O1—C9—H9A	110.4
N2—N1—H1N1	117.5 (13)	C10—C9—H9A	110.4
C6—N1—H1N1	123.0 (13)	O1—C9—H9B	110.4
N1—N2—C7	121.32 (12)	C10—C9—H9B	110.4
C6—C1—C2	118.99 (12)	H9A—C9—H9B	108.6
C6—C1—H1A	120.5	C9—C10—H10A	109.5
C2—C1—H1A	120.5	C9—C10—H10B	109.5
O2—C2—C1	114.90 (12)	H10A—C10—H10B	109.5
O2—C2—C3	123.92 (13)	C9—C10—H10C	109.5
C1—C2—C3	121.17 (13)	H10A—C10—H10C	109.5
C4—C3—C2	118.42 (13)	H10B—C10—H10C	109.5
C4—C3—H3A	120.8	O3—C11—C7	118.97 (13)
C2—C3—H3A	120.8	O3—C11—C12	119.17 (13)
C3—C4—C5	121.74 (13)	C7—C11—C12	121.85 (13)
C3—C4—H4A	119.1	C11—C12—H12A	109.5
C5—C4—H4A	119.1	C11—C12—H12B	109.5
C4—C5—C6	118.40 (13)	H12A—C12—H12B	109.5
C4—C5—H5A	120.8	C11—C12—H12C	109.5
C6—C5—H5A	120.8	H12A—C12—H12C	109.5
C1—C6—C5	121.26 (13)	H12B—C12—H12C	109.5
C1—C6—N1	121.21 (12)	O2—C13—H13A	109.5
C5—C6—N1	117.53 (12)	O2—C13—H13B	109.5
N2—C7—C11	124.15 (12)	H13A—C13—H13B	109.5
N2—C7—C8	114.01 (12)	O2—C13—H13C	109.5
C11—C7—C8	121.84 (12)	H13A—C13—H13C	109.5
O4—C8—O1	122.46 (14)	H13B—C13—H13C	109.5
O4—C8—C7	124.99 (14)		
			
C6—N1—N2—C7	−179.23 (12)	N2—N1—C6—C5	−178.85 (12)
C13—O2—C2—C1	−173.78 (16)	N1—N2—C7—C11	−0.8 (2)
C13—O2—C2—C3	6.6 (3)	N1—N2—C7—C8	178.50 (12)
C6—C1—C2—O2	179.03 (14)	C9—O1—C8—O4	−0.3 (3)
C6—C1—C2—C3	−1.3 (2)	C9—O1—C8—C7	−179.75 (16)
O2—C2—C3—C4	−179.68 (15)	N2—C7—C8—O4	−174.52 (16)
C1—C2—C3—C4	0.7 (2)	C11—C7—C8—O4	4.8 (3)
C2—C3—C4—C5	0.4 (2)	N2—C7—C8—O1	4.92 (19)
C3—C4—C5—C6	−0.9 (2)	C11—C7—C8—O1	−175.78 (13)
C2—C1—C6—C5	0.9 (2)	C8—O1—C9—C10	−178.4 (2)
C2—C1—C6—N1	−178.33 (12)	N2—C7—C11—O3	−3.6 (2)
C4—C5—C6—C1	0.2 (2)	C8—C7—C11—O3	177.20 (13)
C4—C5—C6—N1	179.44 (13)	N2—C7—C11—C12	176.54 (13)
N2—N1—C6—C1	0.4 (2)	C8—C7—C11—C12	−2.7 (2)

### Hydrogen-bond geometry (Å, °)

**Table:**

Cg1 is the centroid of the C1–C6 ring.

**Table:**

*D*—H···*A*	*D*—H	H···*A*	*D*···*A*	*D*—H···*A*
N1—H1N1···O3	0.87 (2)	1.87 (2)	2.5629 (18)	135.3 (18)
C5—H5A···O3^i^	0.93	2.54	3.4389 (19)	164
C13—H13C···O4^ii^	0.96	2.58	3.219 (3)	124
C12—H12B···Cg1^iii^	0.96	2.82	3.6422 (17)	145

Symmetry codes: (i) −*x*+1, −*y*+1, −*z*; (ii) *x*+2, *y*−1, *z*; (iii) *x*−1, *y*+1, *z*.
